# Spatio-temporal patterns of health service delivery and access to maternal, child, and outpatient healthcare in Volta region, Ghana: a repeated cross-sectional ecological study using health facility data

**DOI:** 10.1080/16549716.2025.2513861

**Published:** 2025-06-10

**Authors:** Winfred Dotse-Gborgbortsi, Kristine Nilsen, Ortis Yankey, Anthony Ofosu, Thomas Ankomah, Eric Tweneboah, Ignatius Aklikpe, Chrysantus Kubio, Alberta Biritwum-Nyarko, Andrew Tatem, Jim Wright

**Affiliations:** a WorldPop, School of Geography and Environmental Science, University of Southampton, Southampton, UK; b Department of Social Statistics and Demography, University of Southampton, Southampton, UK; c Department of Community Health, School of Medicine, UHAS, Ho, Ghana; d PPME, Ghana Health Service, Headquarters, Accra, Ghana; e School of Geography and Environmental Science, University of Southampton, Southampton, UK; f Ghana Health Service, Volta Regional Health Directorate, Ho, Ghana

**Keywords:** Stig Wall, Geographic information systems, maternal and child health, healthcare access and utilisation, health management information systems, geographic access

## Abstract

**Background:**

To attain universal health care, health managers need to monitor progress in service uptake, changes and geographic coverage. Although routine health management information systems are now well established in many resource-constrained countries, such data have not yet been used to examine geographic access trends over time.

**Objective:**

This study aims to quantify changing patterns of geographic access to healthcare in the Volta Region, Ghana.

**Methods:**

The repeated cross-sectional ecological spatio-temporal analysis used routine health management information systems data from 2016 to 2022, and geospatial data to examine changes in healthcare accessibility and services provided for population subgroups. Changes in healthcare provision, travel time to services and population coverage were estimated.

**Results:**

Most health facilities (60.6%) provided the same range of services or added new services between 2016 and 2022. Childhood immunisation services had the highest geographic coverage within 30 min of the nearest health facility from 2016 to 2022 (minimum 97.2%), while Caesarean births had the lowest (maximum 75.2%). More health facilities provide antenatal services (2022: 59.9%) than birthing care (2022: 52.6%). Of all new health facilities, 93.2% were Community Health Planning and Services (CHPS) facilities. The majority of the population lived within 30 min of services in 2016 and 2022 for all the services studied.

**Conclusion:**

The study provides a new approach to monitoring service changes through routine health data and spatial analysis. The analysis provided evidence to improve geographic accessibility, address gaps in service changes and consolidate the gains of high geographic coverage with quality care.

## PAPER CONTEXT

- **Main findings**: Primary health care has grown substantially in the study area compared to emergency obstetric care.
- **Added knowledge**: We developed a new process for deducing spatio-temporal changes in service availability with limited routine health data.
- **Global health impact for policy and action**: The results have been used to improve geographic access to healthcare in Ghana, and the method can be replicated in similar settings.

## Background

Sustainable Development Goal (SDG) Target 3.8 seeks to achieve Universal Health Coverage (UHC), including access to quality essential healthcare services for all. The 2023 global UHC progress report estimates only 68% of the world have UHC of essential services for the year 2021 [1]. The UHC index comprises reproductive, maternal, newborn, child health, infectious diseases, noncommunicable diseases, service capacity and access indicators. The African region has the lowest coverage (44%), and Ghana (48%) is only 4% above the regional coverage. While many resource-constrained countries, including Ghana, saw a significant rise in UHC coverage between 2000 and 2015, the growth has plateaued in the SDG era [1].

Aligned with SDG3.8, systematic reviews show that studies have used spatial demographic data, transportation, and health facility databases to assess geographic healthcare access [2,3]. However, most of these studies are cross-sectional and do not consider how spatio-temporal access patterns evolve as the spatial distribution of population changes and facility networks expand or contract [3]. Furthermore, there is limited integration of routine health data with other spatial datasets. Systematic review evidence shows that few studies (3 of 77) applying spatial analysis to maternal and neonatal health in sub-Saharan Africa (SSA) used health management information systems (HMIS) data [2].

Typically, health facilities are classified according to the services that they provide or based on funding models [4]. For example, most facility classifications differentiate tertiary, secondary and primary healthcare, whilst private, charitable and publicly funded facilities are often differentiated [4]. Although several studies have reported persistent staff shortages disrupting service delivery in low- and middle-income countries (LMIC), especially in rural health facilities (Nancy et al. 2021), to date, spatio-temporal facility classification has only been undertaken in high-income countries. For example, a study of the United States of America healthcare facilities used business history and census demographic data to examine spatio-temporal changes in service provision [5]. The business data they analysed contained health facility names, date of establishment, and their active years. Such temporal information might not be available in routine health data or public records in Africa as a recent database of health facilities in Africa did not document when the health facilities became operational [6]. Consequently, relatively few studies of geographic access to healthcare over time consider differences in services targeting specific population sub-groups based on a consistent methodological approach using HMIS [5,7,8]. There are no such studies in LMICs.

HMIS has expanded rapidly in SSA, particularly with the emergence of the District Health Information Software (DHIS2) system as a platform for managing routine health data [9]. In some countries, including Ghana, HMIS use, and data quality auditing have been operational for over 10 years, affording an opportunity to address these knowledge gaps. Integrating HMIS data with spatial demographic data can provide actionable insights for UHC assessment by analysing service availability and access across different populations and regions. This integrated approach enables the identification of service gaps with high spatial resolution, ensuring equitable geographic access to healthcare. It also supports effective planning, monitoring, and resource allocation, while powering evidence-based decisions and interventions.

Therefore, this study aims to use routine HMIS, demographic, and ancillary geospatial data to quantify changing patterns of geographic access to healthcare in the Volta Region, Ghana. In doing so, it aims to assess whether trends in geographic healthcare access vary by population sub-group, specifically between mothers and children under 5 years, relative to the general population. Secondly, it aims to develop a spatio-temporal classification of healthcare facilities based on reported changes in service delivery over time.

## Methods

### Study design and area

**Figure 1.**
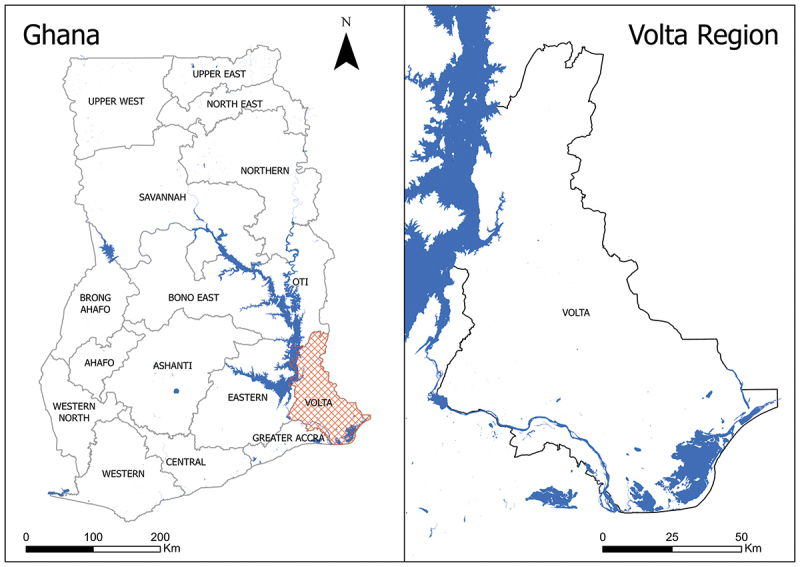
Location of Volta Region in Ghana.

The design is a repeated cross-sectional study conducted in the Volta Region of Ghana, using routine health facility data. The Volta Region, one of Ghana’s 16 regions, was chosen because there is sufficient geographic data on health facility locations. The Volta Region is located in South Eastern Ghana with approximately 1.65 million inhabitants (Figure 1). The region covers approximately 9,504 km^2^ with 174 persons km^−2^ [10]. The region shares boundaries with Lake Volta (one of the world’s largest man-made lakes) to the west and the Atlantic Ocean to the south, making fishing a predominant economic activity at these locations and creating island communities that are hard to reach.

The Community-Based Health Planning and Services (CHPS) policy was implemented nationwide in Ghana since 2002 to provide a minimum package of basic treatment of illnesses, nutrition, maternal and child health at the community level [11]. The CHPS initiative mobilised communities and stakeholders to set up health facilities within the community with residences for trained health workers to serve the catchment population. CHPS aims to improve access to services and meet community health needs. Each CHPS facility is managed by a Community Health Officer or a midwife with support from health centres that manage sub-district service provision. Physician Assistants and midwives usually lead health centres. CHPS and health centres refer complicated cases to hospitals where medical doctors are located for advanced care [12]. Maternity homes provide birthing care and are usually operated privately by qualified midwives. Health facilities can be public, private, faith-based, or government-assisted. To reduce the financial burden of using services, a health insurance scheme was introduced in 2003 to limit out-of-pocket payments [13].

## Data sources and selection of health facilities

The web-based District Health Information Management System v2 (DHIMS2) was implemented in Ghana in 2012 to replace the standalone MS-Access DHIMS2 database. Ghana’s DHIMS2 is developed using the DHIS2 source code [14]. Service providers and health information officers routinely input aggregated health data into DHIMS2 after collating service data from paper registers. The DHIMS2 system collects data from all health system levels, including public and private health facilities [15]. Table 1 shows the characteristics of the health facility and geospatial datasets used in this study.

**Table 1.** Coverage, resolution and purpose of data sets used for the study.

Map layer	Source	Temporal coverage & resolution	Spatial resolution	Purpose
Health facility Service information and geographic location	Ghana Health Service [15]	2016 to 2022	n/a	Analyse the location of health facilities and their geographic coverage
District and sub-district boundaries	Ghana Health Service [15]	2022	The average district size is 556 square kilometres The average sub-district size is 107 square kilometres	Validate health facility locations.
Digital Elevation Model	Shuttle Radar Topographic Mission [16]	2000	90 meters	Moderate walking speed in travel time model
Land cover	European Space Agency [17]	2021	10 meters aggregated to 100 meters	Model travel time
Roads	OpenStreetMap [18]	As at 1st November 2023	Vector data converted to 100 meters raster	Model travel time, enables more rapid travel
Water bodies (rivers and lakes)	OpenStreetMap [18]	As at 1st November 2023	Vector data converted to 100 meters raster	Model travel time, serves as a travel barrier
Population	WorldPop [19]	2016 to 2022	100 meters	Estimate population coverage by travel time

All health facilities in the Volta region were included in the study. We analysed health data aggregated by health facility and year from 2016 to 2022 for the Volta Region. As DHIMS2 data quality and completeness have improved over time since its inception in 2012, we used 2016 as a baseline for the seven-year period, as this period is likely to have better data [20].

## Health service indicators examined

We selected six service indicators: outpatient attendance, number of people receiving malaria treatment, number of antenatal registrants, number of women giving birth, number of caesarean births and number of children receiving the first dose of pentavalent vaccine. These service indicators were chosen to represent the general population (outpatient attendance and malaria care), women of childbearing age (WoCBA) (antenatal, skilled birthing care and caesarean care for obstetric emergencies), and children under 5 years (pentavalent vaccine).

The selected indicators capture treatment (malaria and caesarean care), preventive (pentavalent vaccine), and health promotion services (antenatal and birth care) [21]. These indicators align with standardised frameworks for UHC assessment [21]. Furthermore, they are established tracers for monitoring SDG Goal 3.8 on access to essential health services and vaccines, as demonstrated in global studies [22] and validated in local settings similar to ours [23].

## Health facility locations

The Ghana Health Service also provided the geographic location of health facilities in the Volta Region. The health facility locations were examined for accuracy. We spatially joined health facility locations and district administrative boundaries from the Ghana Health Service to check if health facilities were contained in their coordinating administrative districts. Examination of the non-matching health facilities showed some errors. The longitude and latitude of some health facilities were swapped, and the decimals of some coordinates were wrongly placed or missing some digits. Some health facilities lay in another district but close to their coordinating administrative district. These errors in health facility locations were corrected. Furthermore, health facilities near but not completely within their district boundary were compared to reference place name data from the Ghana Statistical Service. When a place name and corresponding health facility are co-located but outside their district administrative boundary, the boundaries were considered inaccurate, and the health facility locations remain unchanged.

## Temporal trends in service provision

We calculated the percentage of health facilities providing outpatient, malaria treatment, antenatal, birthing care, caesarean births, and childhood vaccination services (using the first dose of pentavalent vaccine as proxy) from 2016 to 2022. A facility provides a service in a year if the aggregated number of persons receiving the service is greater than zero. The percentage of health facilities providing a service is calculated by dividing the number of health facilities providing a given service in a year by the total number of operational health facilities. Also, we calculated the changes in the percentage of health facility types (CHPS, health centre, hospital, and maternity homes) providing each of the six services, except caesarean care, from 2016 to 2022. Only hospitals were considered for assessing caesarean birth coverage. The numerator is the number of facilities of a given type that provided services in a year divided by the total number of that health facility type operational in that year (e.g. the number of CHPS providing antenatal care in 2016 divided by the total number of operational CHPS facilities).

## Classification of health facilities

A health facility is considered to have provided a service if it reported any count of persons receiving the service in a particular year in DHIMS2. The health facilities were classified into five functional service provision groups: closed, newly operational, no service, oscillating and existing.

- Closed health facilities provided at least one service in 2016 but no service in 2022.
- Newly operational health facilities were providing no service in 2016 but at least one service in 2022.
- Facilities were designated ‘no service’ if they provided no services in the 7 years from 2016 to 2022.
- Oscillating health facilities provided services in some years from 2017 to 2021 but no services in 2016 and 2022.
- Health facilities grouped as ‘existing’ provided at least one service each year from 2016 to 2022. The existing health facilities group was sub-categorised into increased, decreased, unchanged or varying services. ‘Unchanged existing’ health facilities provided the same combination of services throughout the study period. ‘Existing increased’ health facilities provided a greater range of services in 2022 than in 2016. In contrast, health facilities with more services in 2016 than in 2022 were classified as ‘decreasing’. Services are categorised as ‘varying’ if services provided in 2016 and 2022 are the same, but there are variations for the years in between.

We tested the association between a health facility ownership/funding model (private, public and faith-based) and service functionality with a Fisher's exact test.

## Travel time analysis

We used a cost distance approach implementing a multimodal walking and mechanised travel time model as previously undertaken in Ghana [24] and elsewhere [25]. The cost distance travel time model accumulates the time it takes to reach the nearest health facility through the least cost path of 100 m grid cells. Travel speeds from tracked roads in the nearby Eastern Region were applied to roads in the Volta Region [26]. Rivers and lakes were considered obstacles to travel. Walking speeds were moderated using slopes derived from elevation data and Tobler’s formula as applied in a previous study [25]. Travel times were estimated for health facilities providing each of the six services considering the changes from 2016 to 2022. For each service, we calculated the difference between 2016 and 2022 to derive the change in travel time. We mapped the travel time estimates for 2016 and 2022 and the change between the 2 years.

## Population projection

We used the 2021 population census data for Ghana [27], disaggregating these census data to 100 m grid cells using a random forest dasymetric population disaggregation approach [19]. We used the 2021 disaggregated population as a baseline estimate and projected the 2021 population numbers using the annual percentage growth rate provided by the World Bank [28]. We implemented forward projections to obtain 2022 gridded population estimates and back-projections to obtain gridded population estimates for 2018 to 2020 using a simple arithmetic projection. The forward-projected population was based on the formula: $P_{n}=P_{0}\left(\frac{r}{100}\right)$ where $P_{n}$ is the expected population for 2022, $P_{0}\;$ is the baseline population for 2021, and *r* is the population growth rate. The backwards-projected population for years 2018 to 2020 was based on the formula $P_{n}=\frac{P_{0}}{\left(r/100\right)}$ where $P_{n}$ is the expected population for the various years 2018 to 2020, $P_{0}\;$ is the baseline population for 2021 and *r* is the population growth rate. We also estimated age-sex proportions for each projected population using age-sex population proportions derived from the 2021 census.

Relevant age-sex grid cells were summed to derive the population sub-groups for children under 5 years, WoCBA, and the total population.

## Estimating geographic coverage with travel time and projected population

We classified the travel times into 30 min, 31–60 min, 61–120 min and more than 120 min, as women with obstetric complications could die if they do not reach a well-equipped health facility within 2 hours [29]. Beyond maternal health, these travel time groups are relevant for other population sub-groups to estimate geographic coverage within reasonable travel time limits. For each of the six health service indicators and their corresponding population sub-groups, we estimated the percentage of persons living within the travel time groups for 2016 and 2022. The annual population coverage results are presented on maps and trend plots.

## Results

There were 559 health facilities recorded in DHIMS2 in the Volta Region in 2022. Most were CHPS 397 (71.0%) and health centres 117 (20.9%) (Table 2). There were 34 (6.1%) hospitals and 11 (2.0%) private maternity homes. Of the 559 health facilities, 85% were public, 13.6% private, and 1.4% faith-based. Most private health facilities were CHPS (65.8%) and hospitals (19.7%). Among government health facilities, CHPS (72%) and health centres (24.6%) predominate.

**Table 2.** Percentage distribution of health facility types by ownership, Volta region, 2022.

Facility Type	Facility Ownership			
	Government Frequency (%)	Faith-based Frequency (%)	Private Frequency (%)	Total Frequency
CHPS	344 (86.6)	3 (0.8)	50 (12.6)	397
Health Centre	117 (100.0)	0 (0.0)	0 (0.0)	117
Hospital	14 (41.2)	5 (14.7)	15 (44.1)	34
Maternity Home	0 (0.0)	0 (0.0)	11 (100.0)	11
Total	475 (85.0)	8 (1.4)	76 (13.6)	559

## Changes in healthcare provision

**Figure 2.**
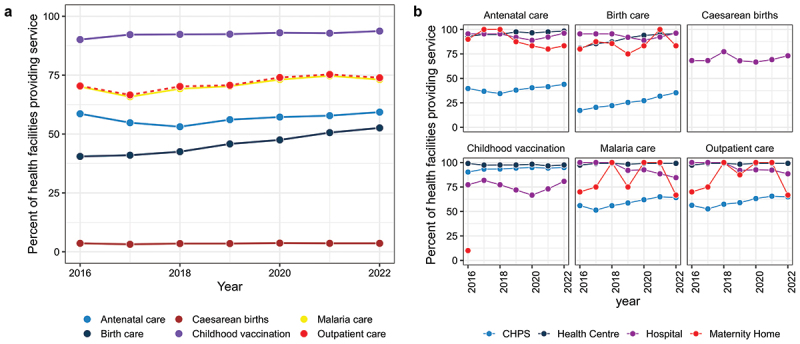
Temporal changes (2016 to 2022) in: A. The percentage of health facilities, among those operational in a given year, providing outpatient, malaria, antenatal, birth, caesarean, and childhood vaccination care in the Volta Region. B. The percentage of health facilities providing services by health facility type in the Volta region, among those operational in a given year.

The proportion of health facilities (among those operational in a given year) providing each service is presented in Figure 2a. Most health facilities provided childhood vaccination services, while few offered caesarean care. Outpatient services and malaria care were the second-highest services. There was almost a perfect positive correlation between the proportion of health facilities providing outpatient and malaria care across the years. Antenatal services and birthing care provision for pregnant women were lower than outpatient services and childhood vaccination. Also, there was a positive correlation between antenatal and birthing care, although antenatal care was more prevalent than birthing care in operational health facilities.

Figure 2b disaggregates the proportion of health facilities providing each service by health facility type. In 2016, 39.6% of CHPS provided antenatal services, compared to 17.2% with birthing care services. Generally, the proportion of health facilities providing antenatal care decreased between 2016 and 2018 but increased steadily thereafter. Antenatal and birthing care increased in 2022 to 43.9% and 35.5%, respectively, among CHPS. In private maternity homes, antenatal care provision decreased while birthing care increased. Childhood vaccination, malaria care and outpatient services increased in CHPS facilities but plateaued in health centres and hospitals. Almost all health centres provided antenatal care, outpatient services and malaria care. Although 80.9% of health centres provided birthing care in 2016, it increased to 95.8% in 2022. Most of the six services were provided in hospitals. However, there were decreases in malaria and outpatient services among private hospitals.

**Figure 3.**
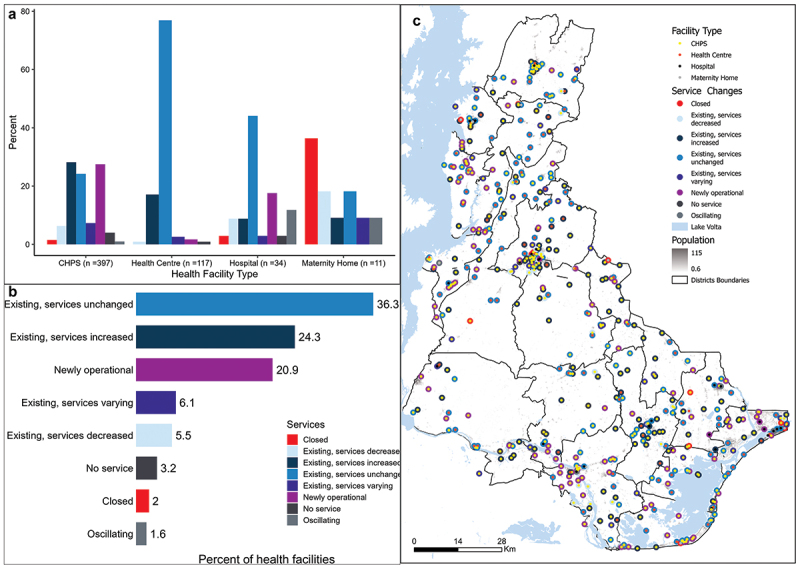
A. Change in services provided by health facility type. B. Percentage distribution of the changes in health facilities C. Spatial distribution of health facility types and changes in services provided overlayed on 2022 population.

Between 2016 and 2022, the most newly operational health facilities (117) were CHPS (93.2%) and hospitals (5.1%) (Figure 3a). Of the six newly operational hospitals, five were private and one was a government hospital in Ketu North District, which became operational in 2022. Most newly operational facilities (43.6%) provide childhood vaccination services. However, service provision in private health facilities was the most unstable. The results show that 7 (77.8%) of the 9 oscillating and 10 (90%) of the facilities that closed down were private. Similarly, the absolute number of private maternity homes providing birthing care decreased from eight in 2016 to five in 2022, reducing by one each year.

Most public health centres (76.9%) and many hospitals (44.1%), remaining unchanged, have provided the same combination of services over the study period. CHPS and health centres have increased the number of services they provide compared to other health facility types. Among the 18 facilities in the DHIMS2 database with no data for the six services in the study period, 10 are private and eight are public. Of these 18, 16 were CHPS, one was a private hospital and one was a public health centre. Generally, 60.6% of health facilities have remained unchanged or increased their number of services. There were 20.9% newly operational facilities (Figure 3b). However, newly operational CHPS facilities and unchanged health centres are dominant in most districts. There is a statistically significant association between health facility funding model or ownership and their functionality (*p* < 0.001). On average, health centres and hospitals provided the highest number of services. The spatial distribution of health facilities does not show any unusual patterns (Figure 3c).

## Trends in geographic healthcare access

**Figure 4.**
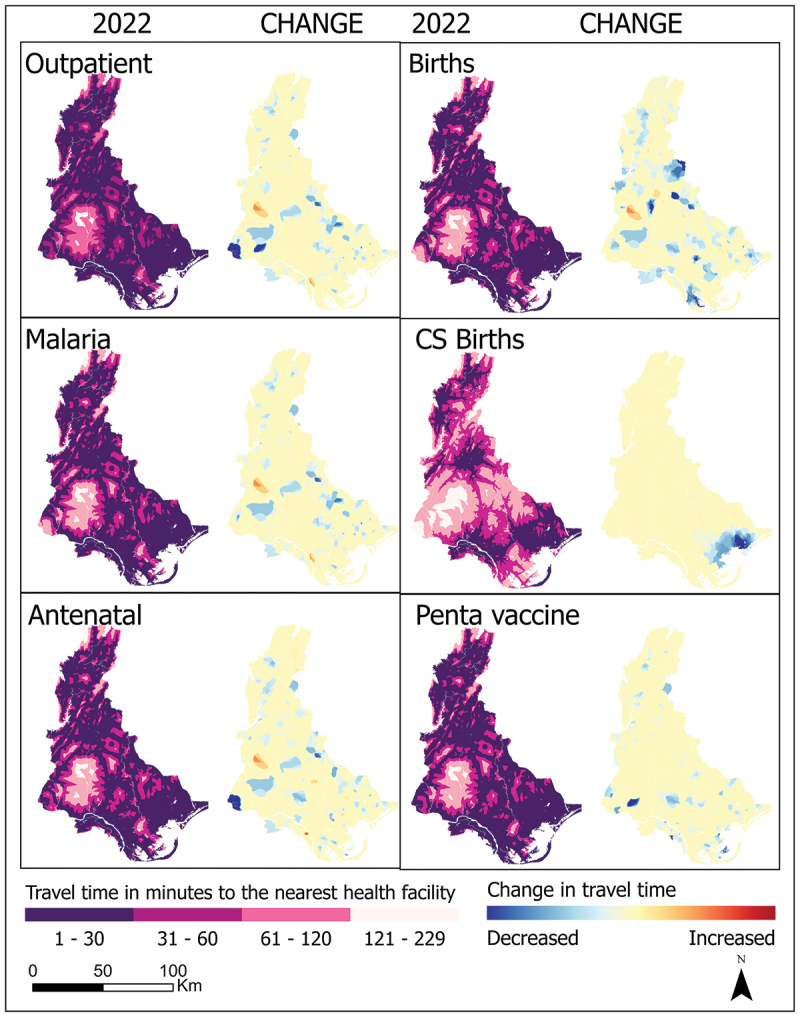
Change in travel time between 2016 and 2022 for facilities reporting outpatient, malaria, antenatal, birth, caesarean, and childhood vaccination care, Volta Region.

Most areas in the Volta region were within 30 minutes’ travel to the nearest health facility for most services except caesarean births, as shown in Figure 4. Most parts of the region show no changes in travel times. However, outpatient services, malaria care, antenatal care and birthing services show the most changes, with generally decreasing travel time to the nearest health facility. Vaccination services and caesarean births showed no increase in travel time, while the other services observed small pockets of negative change. While the other services underwent region-wide changes, changes in caesarean birth coverage were limited to southeastern Volta.

## Population changes

**Figure 5.**
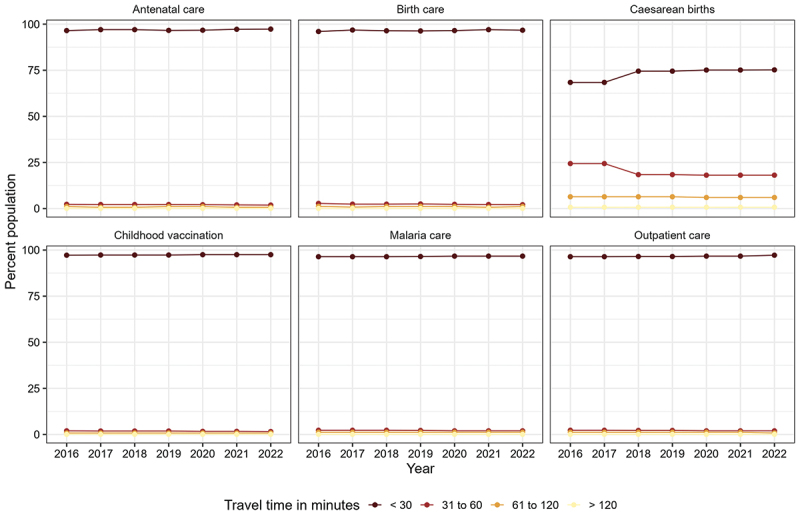
Population coverage change between 2016 and 2022 in the Volta Region for outpatient, malaria, antenatal, birth, caesarean, and childhood vaccination care.

Figure 5 shows the percentage of the population near health facilities. In 2016, all the services covered at least 96% of their target population, except caesarean births (68.4%). Caesarean care coverage within 30 min travel ranged from 68.4% in 2016 to 75.2% in 2022. Geographic coverage of caesarean birth services within 30 minutes’ travel time increased in 2018 and was relatively unchanged thereafter. The other services saw only a marginal increase in population coverage as their geographic coverage was relatively high.

## Discussion

In this paper, we present the first analysis of spatio-temporal changes in health services availability and coverage using HMIS data in SSA. Our results showed high geographic accessibility within 30 min for most of the services, but there were differences in coverage by population group. Child vaccination services had the greatest coverage, mainly through CHPS facilities, while health facilities with surgical capacity for caesarean births had the lowest geographic coverage. The number of health facilities providing services, particularly CHPS, increased during the study period, but private facilities were unstable or underwent closures.

The patterns observed can be partially explained by the type of service, policy, infrastructure, and human resource availability. In Ghana, only hospitals are equipped with the infrastructure and human resources to perform caesarean births due to the complexity of the procedure. Immunisation services are most prevalent, with the highest coverage, because they are the only service among the ones we studied that provides services at the health facility and via community outreach points. Thus, there are fewer missed opportunities for immunising children as outreach services decrease service user travel times and increase immunisation coverage [30]. Before midwives complement existing staff to provide birthing care, community health officers usually treat minor illnesses such as malaria and diarrhoea in newly established health facilities, particularly CHPS. This might explain the almost perfect correlation between outpatient services and malaria treatment. The impact of CHPS on increasing access to maternal and child health services in Ghana is evident in our analysis and others [31,32].

Antenatal and birthing care could be highly correlated because multiple antenatal care appointments promote health facility births in Ghana (Dotse-Gborgbortsi et al. 2020). Both services are usually provided in health facilities by a midwife, nurse, or doctor. However, the number of health facilities providing antenatal care is more than birthing care providers because some trained community nurses provide antenatal care in CHPS facilities but are unable to assist with births. Another plausible reason is that some midwives visit CHPS facilities to provide antenatal care, but the women must travel to the health centre for birthing care. The mismatching antenatal versus birthing service provision could explain the low continuity of care at the place of delivery in the Volta Region, as many pregnant women switched providers [33]. Meanwhile, women who stay with the same provider through the maternity care path report are more satisfied [34].

The data suggest that over the study period, new health facilities usually become established through the following stages: communities initially receive immunisation services through outreach before a health facility is set up to treat minor illnesses. Then, a midwife joins the existing staff to provide antenatal and birthing care. Regardless, some trained community health nurses offer antenatal care in CHPS facilities by themselves or through visiting midwives. This pattern observed in the data is consistent with the CHPS policy guidelines [11].

Hospitals with surgical care and staff ready to assist caesarean births are expensive to build and run. Therefore, considering the resource constraints in the study area, few health facilities provide caesarean services, and few were established during the study period. While many health centres and CHPS were established in the Volta region alone during the study period, the Government’s plan to build 111 hospitals across the nation (8 in the Volta Region) has not yet been completed since the project was launched in 2021 [35]. When built, these eight new hospitals will substantially increase geographic access to surgical care for complicated obstetric cases.

One key finding is the decline of private health facilities, including maternity homes, in the region. There was a statistically significant association between health facility ownership and functionality. Ensuring that all health facilities, including all the private ones, will enable improved oversight and holistic joined-up management of public and private providers. Data from all private providers will facilitate enhanced geographic coverage analysis for monitoring UHC.

The decline in private maternity homes is a worrying trend as they provide birthing care services, especially in underserved rural areas. The declining trend is not new in Volta and other regions in Ghana. In 2014, a project aimed at saving maternity homes in six regions, including the Volta Region, because 100 private maternity homes were closed in the 5 years before the project [36]. The project report cited ageing midwives and delayed health insurance reimbursement as the key reasons private maternity homes were closing [36]. Also, midwives who have retired from the government health facilities operate these private maternity homes and usually do not have succession plans.

The distribution and growth of services could have several implications for health outcomes. There is substantial growth in the number and geographic coverage of health facilities with immunisation services in the Volta Region. We found 97.5% of the children 5 years or less living within 30 min of a health facility providing immunisation services in 2022. The 2022 Ghana Demographic and Health Survey (DHS) results show 80% of children 12–23 months in the Volta Region were fully immunised, and 97% of eligible children received the first dose of the pentavalent vaccine [37]. The DHS and our study findings imply that the high geographic coverage of immunisation services could account for the correspondingly high proportion of children fully vaccinated in the Volta Region. Furthermore, the higher proportion of health facilities providing antenatal care than skilled birth services in the region is also reflected in the DHS survey results, with antenatal care coverage higher at 99.2% compared to 90.9% for health facility births [37].

Some women might not be accessing institutional birthing care due to the longer travel times to reach health facilities offering such services [3]. However, timely access to quality comprehensive emergency obstetric care in emergencies is critical for preventing maternal mortality [38]. Caesarean care is a key component of comprehensive emergency obstetric care, but it is the most geographically limited service in the region and has not seen substantially increased geographic coverage relative to population growth. Thus, many women will have to travel across districts with poor roads and limited ambulances to receive comprehensive emergency obstetric care. To mitigate the potential impact of low geographic access to comprehensive obstetric care, referral systems from CHPS and health centres to hospitals need strengthening. The poor transportation and referral networks have to improve to ease the burden of accessing critical emergency care, especially for rural communities [39].

Although more than 90% of the women could reach a health facility within 2 hours, similar to other sub-Saharan African settings [40], the time window might be too large for some obstetric emergencies. Coupled with the low geographic coverage of surgical care, some district hospitals do not have sufficient human resources and infrastructure to provide quality surgical care [41], which could lead to avoidable deaths [42].

Ghana’s progressive CHPS initiative is similar to the Health Extension Program in Ethiopia, Integrated Community Case Management in Senegal and other community health initiatives in Africa [43]. Although there are challenges, primary health policies to promote UHC in SSA have improved maternal and child health through health extension workers [44] and increased access to treatment for basic illnesses like malaria [45]. To strengthen the community health systems in SSA, financing, logistics, community ownership and other challenges need addressing [46].

Spatio-temporal health facility classification is relevant to health service delivery. Geographic coverage in sub-national administrative areas can be ranked to target interventions. For example, the gap between antenatal and birthing care coverage can be decreased by placing midwives in all health facilities with antenatal care and providing an enabling environment for them to provide services. The patterns can reveal how services are onboarded for newly operational health facilities, as seen in our study. Furthermore, repeated analysis could help identify health facilities that need support, such as those closing, oscillating, or regularly with varying service availability. However, services must improve in quality to consolidate the high geographic coverage. For instance, a study in the Volta Region found high immunisation dropout rate and reduced antenatal care utilisation as challenges to attaining UHC [47]. Such gaps must be identified and addressed to ensure geographic coverage is accompanied by high-quality service.

Our study approach is unique in classifying the spatiotemporal changes in health facilities. It can identify new health facilities and the services they provide over time, provided those services are offered to reasonably large patient populations. The indicators and period were carefully chosen to avoid potential data quality issues. The services we chose are frequently used, so we are unlikely to observe zero case counts. Also, the data was aggregated yearly for each service so that even if there was non-reporting in a certain month, it would have less effect on the whole year. The study area can be expanded to include the rest of Ghana, as there are HMIS and health facilities nationwide. Furthermore, other key services such as adolescent health, sexual and reproductive services, child growth monitoring and other key indicators could be included in future studies.

Due to the unique health systems and distribution of health facilities, our findings might not apply to other settings. However, large-scale studies in Sub-Saharan Africa can in principle assess spatio-temporal service changes, given extensive DHIS2 adoption across the region as system use matures [9]. Our workflow can be extended by integrating other datasets and indicators collected via HMIS to monitor UHC progress.

Data quality can be a concern when analysing HMIS data. The errors associated with the geographic location of health facilities can affect the travel time estimates and population coverage analysis. The functionality of some health facilities could be misclassified due to data quality. For instance, non-reporting, particularly among private health facilities, could lead to misclassification. Also, the classification could be sensitive to the 2016 base year. For instance, a health facility which was operational but closed in 2016 and reopened later could be misclassified as we did not analyse prior years. We did not account for the possibility of people from nearby regions using services in the Volta Region, which could moderate the effect of bypassing health facilities, although there is cross-regional use of services [26]. Some assumptions around travel time modelling may affect the estimates. Firstly, land cover and road conditions might sometimes change during the study period due to rainfall or flooding [48]. Likewise, people could use other forms or combinations of travel modes rather than the multimodal one we implemented. Also, transportation costs could limit some women from accessing health facilities.

## Conclusion

To attain UHC, health managers must constantly monitor service availability, utilisation, quality, and geographic coverage progress. Given the paucity of historical data describing health facilities until the recent growth in the availability of HMIS in resource-constrained settings, we measured spatio-temporal changes in service availability in Ghana. This study highlights the utility of routine health data and developed an approach to measuring geographic coverage and changes in service availability by building on the growing temporal archive of HMIS data. While providing essential information for service improvement in Ghana, it also develops the blueprint for future studies with HMIS data.

## Supplementary Material

STROBE_completed.doc

## Data Availability

The routine health data, health facility coordinates and administrative boundaries analysed are not publicly available due to confidentiality and data licencing restrictions from the Ghana Health Service. They can be obtained from the Ghana Health Service (https://www.ghs.gov.gh/contact-us) with reasonable request The digital elevation data are available from the USGS via Earth Explorer (https://earthexplorer.usgs.gov/), doi: https://doi.org/10.5066/F7PR7TFT The land cover data is available from the European Space Agency (https://worldcover2020.esa.int/download) doi: https://doi.org/10.5281/zenodo.5571936 Road networks and water bodies spatial data are freely available from the OpenStreetMap and its contributors (https://www.openstreetmap.org/) via Geofabrik (https://www.geofabrik.de/) Gridded population datasets are freely available from the WorldPop Research Group at the University of Southampton (https://www.worldpop.org/datacatalog/)

## Preprint

This study is available as a preprint on Research Square. The details are: Dotse-Gborgbortsi W, Nilsen K, Yankey O, Ofosu A, Ankomah T, Tweneboah E, et al. Spatio-temporal patterns of health service delivery and access to maternal, child, and outpatient healthcare in Volta Region, Ghana: a repeated cross-sectional ecological study using health facility data. Research Square Preprint. 2025. doi: 10.21203/rs.3.rs -6,195,222/v1.
