# Supplementary material for: Spatio-temporal patterns of health service delivery and access to maternal, child, and outpatient healthcare in Volta region, Ghana: a repeated cross-sectional ecological study using health facility data
Source: Glob Health Action. 2025 Jun 10;18(1):2513861. doi: 10.1080/16549716.2025.2513861 (PMC12152985; doi:10.1080/16549716.2025.2513861)
Supplement: STROBE_completed.doc [file ZGHA_A_2513861_SM5405.doc]

STROBE Checklist: Spatio-temporal patterns of health service delivery and access to maternal, child, and outpatient healthcare in Volta Region, Ghana: a repeated cross-sectional ecological study using health facility data

|  | Item No | Recommendation | Comments |
| --- | --- | --- | --- |
| **Title and abstract** | 1 | (*a*) Indicate the study’s design with a commonly used term in the title or the abstract | The title and abstract clearly state that the study design is a "repeated cross-sectional ecological study," commonly used to describe the study approach. |
|  |  | (*b*) Provide in the abstract an informative and balanced summary of what was done and what was found | The abstract includes a balanced summary of the study's objectives, methods (using routine health facility data and geospatial models), and key findings (including changes in health service coverage over time and geographic access). |
| Introduction | | |  |
| Background/rationale | 2 | Explain the scientific background and rationale for the investigation being reported | The introduction provides a clear rationale for using a novel, scalable approach to examine changes in health service coverage through routine health data in resource-limited settings like the Volta Region, Ghana. |
| Objectives | 3 | State specific objectives, including any prespecified hypotheses | The study's objectives are explicitly stated, focusing on quantifying the geographic access to healthcare and changes in services provided in the Volta Region by analysing routine health data, demographic data, and geospatial data. |
| Methods | | |  |
| Study design | 4 | Present key elements of study design early in the paper | Our study design - repeated cross-sectional study – is the first line of the methods section on page 5 under the subtitle “Study design and area”. |
| Setting | 5 | Describe the setting, locations, and relevant dates, including periods of recruitment, exposure, follow-up, and data collection | The study setting, including geographic details and a map (Figure 1), is clearly described. The study period (2016–2022) and the Volta Region's relevance are well-justified.  We did not conduct interviews so there was no recruitment, exposure, and follow-up. |
| Participants | 6 | (*a*) Give the eligibility criteria, and the sources and methods of selection of participants | We stated that “All health facilities in the Volta region were included in the study” under Data sources sub section.  Our secondary data sources and characteristics are described in Table 1. |
| Variables | 7 | Clearly define all outcomes, exposures, predictors, potential confounders, and effect modifiers. Give diagnostic criteria, if applicable | Health service indicators (e.g., outpatient attendance, malaria treatment, maternal health services) are clearly defined in the Methods section. As this is an ecological study, individual-level predictors and effect modifiers were not examined. |
| Data sources/ measurement | 8* | For each variable of interest, give sources of data and details of methods of assessment (measurement). Describe comparability of assessment methods if there is more than one group | The data sources are well-described, with a table (Table 1) outlining the characteristics of health facility data, geographic data, and service indicators. The methods of data collection are well-documented, ensuring transparency. |
| Bias | 9 | Describe any efforts to address potential sources of bias | As stated in the discussion, we purposefully chose high-volume health services and used yearly aggregates to limit zero reporting and the effect of missing data on our analysis procedure. |
| Study size | 10 | Explain how the study size was arrived at | All health facilities in the Volta Region were included in the analysis, ensuring a comprehensive study without the need for sampling. |
| Quantitative variables | 11 | Explain how quantitative variables were handled in the analyses. If applicable, describe which groupings were chosen and why | Quantitative variables such as travel time to health facilities were classified according to World Health Organisation recommendations. The methods for calculating these variables (e.g., multimodal walking and mechanised models) are clearly described. |
| Statistical methods | 12 | (*a*) Describe all statistical methods, including those used to control for confounding | Descriptive statistics were used throughout the analysis. No statistical models were applied as the study relied on secondary data from health facilities, and geospatial modelling was employed for travel time analysis. |
|  |  | (*b*) Describe any methods used to examine subgroups and interactions | Not applicable as we did not construct any statistical model |
|  |  | (*c*) Explain how missing data were addressed |  |
|  |  | (*d*) If applicable, describe analytical methods taking account of sampling strategy | Our analysis included all eligible health facilities so we did not have to account for sampling. |
|  |  | (*e*) Describe any sensitivity analyses | Not applicable as we did not construct any statistical model |
| Results | | |  |
| Participants | 13* | (a) Report numbers of individuals at each stage of study—eg numbers potentially eligible, examined for eligibility, confirmed eligible, included in the study, completing follow-up, and analysed | All health facilities in the Volta Region were included in the analysis, with no attrition or loss to follow-up as this is a secondary data study. |
|  |  | (b) Give reasons for non-participation at each stage | This is a secondary data analysis that included all eligible health facilities. Therefore, there was no recruitment and non-participation. |
|  |  | (c) Consider use of a flow diagram | There was no need for a flow diagram to illustrate participation. |
| Descriptive data | 14* | (a) Give characteristics of study participants (eg demographic, clinical, social) and information on exposures and potential confounders | Descriptive statistics, including the percentage distribution of health facilities, are presented in Table 2. |
|  |  | (b) Indicate number of participants with missing data for each variable of interest | Data on missing values are not applicable as there was no missing data. |
| Outcome data | 15* | Report numbers of outcome events or summary measures | Outcome data are clearly reported with summary statistics, including percentage changes in service provision and geographic and population coverage for the study period.. |
| Main results | 16 | (*a*) Give unadjusted estimates and, if applicable, confounder-adjusted estimates and their precision (eg, 95% confidence interval). Make clear which confounders were adjusted for and why they were included | No statistical models were applied, but descriptive trends over time for health services provided, geographic access, and population coverage are clearly presented. |
|  |  | (*b*) Report category boundaries when continuous variables were categorized | Category boundaries were reported for travel time groupings. |
|  |  | (*c*) If relevant, consider translating estimates of relative risk into absolute risk for a meaningful time period | Not applicable as we did not estimate risk. |
| Other analyses | 17 | Report other analyses done—eg analyses of subgroups and interactions, and sensitivity analyses | Not applicable |
| Discussion | | |  |
| Key results | 18 | Summarise key results with reference to study objectives | Key results are summarized and interpreted in the context of the study objectives, with an emphasis on changes in healthcare service coverage and geographic access. |
| Limitations | 19 | Discuss limitations of the study, taking into account sources of potential bias or imprecision. Discuss both direction and magnitude of any potential bias | The limitations of the study, including data quality issues, classification challenges for health facilities, and potential edge effects in the spatial analysis, are discussed in the manuscript. The lack of seasonality in the travel time model is also acknowledged. |
| Interpretation | 20 | Give a cautious overall interpretation of results considering objectives, limitations, multiplicity of analyses, results from similar studies, and other relevant evidence | The interpretation of results is cautious, considering the study's limitations, such as potential biases in secondary data and the ecological nature of the analysis. |
| Generalisability | 21 | Discuss the generalisability (external validity) of the study results | The generalisability of results is discussed, highlighting that while findings may not be directly applicable to other settings, the methodology can be adapted to similar low-resource settings using routine health data. |
| Other information | | |  |
| Funding | 22 | Give the source of funding and the role of the funders for the present study and, if applicable, for the original study on which the present article is based | Funding sources are clearly stated in the manuscript. |
